# Systematic review on health care for transvestites and transsexuals in Brazil

**DOI:** 10.11606/s1518-8787.2023057004693

**Published:** 2023-03-29

**Authors:** Rafael Rodolfo Tomaz de Lima, Taiana Brito Menêzes Flor, Luiz Roberto Augusto Noro

**Affiliations:** I Universidade Federal do Rio Grande do Norte Centro de Ciências da Saúde Programa de Pós-Graduação em Saúde Coletiva Natal RN Brasil Universidade Federal do Rio Grande do Norte. Centro de Ciências da Saúde. Programa de Pós-Graduação em Saúde Coletiva. Natal, RN, Brasil

**Keywords:** Transvestism, Transsexualism, Minority Health, Unified Health System, Systematic Review

## Abstract

**OBJECTIVE:**

To synthesize scientific evidence to characterize health care for transvestites and transsexuals in Brazil.

**METHODS:**

This is a systematic review, conducted from July 2020 to January 2021 and updated in September 2021, whose protocol is registered in the International Prospective Register of Systematic Reviews (PROSPERO) platform, under code CRD42020188719. The survey of evidence was carried out in four databases and eligible articles were evaluated for methodological quality, and those with a low risk of bias were included.

**RESULTS:**

Fifteen articles were selected and the findings were grouped into six categories according to their thematic approaches: Possibilities to transform health care; Transvestiphobia and transphobia: violations inside and outside the Brazilian Unified Health System (SUS); Professional unpreparedness to care for transvestites and transsexuals; Search for health care alternatives; Right to health for transvestites and transsexuals: utopia or reality?; The Transsexualization Process: advances and challenges.

**CONCLUSIONS:**

There is evidence that health care for transvestites and transsexuals in Brazil is still exclusive, fragmented, centered on specialized care and guided by curative actions, resembling the care models that preceded the SUS and which have been heavily criticized since the Brazilian Sanitary Reform.

## INTRODUCTION

Transgender identities are diverse and not limited to a specific definition. However, in this study, transgender people are understood as those who have a gender identity opposite to the sex assigned at birth^1^. In addition, the term transvestite precedes the term transsexual and is more frequent in Brazil and in other Latin American countries, designating people who experience female roles, but who do not recognize themselves as men or women^1^.

In the global context, transvestites and transsexuals represent a small portion of the general population. However, the discrimination and social exclusion imposed on these people put them in the worst health and life conditions^3^. Compared to other population groups, transvestites and transsexuals have high rates of mental suffering, including suicidal ideation and attempts, due to the discrimination and rejection they face throughout their lives and in all institutional spaces. In addition, other health problems are caused, mainly, by bodily transformations resulting from attempts to align the phenotype with gender identity. Thus, these factors end up generating a lower life expectancy and greater difficulties for these people to access health services^3 , 4^.

In Brazil, similar to the reality of other countries, transvestites and transgender women are socially more vulnerable: among lesbians, gays, bisexuals, queers, intersexuals, asexuals and other categories of gender and sexuality (LGBTTQIA+), they are the main victims of violence, especially bodily injuries and homicides by firearms^5 , 6^. Given this reality, health care should not be configured only in assistance to this population’s health problems, but also in broad and concrete actions to welcome transvestites and transgender people, which help them face gender identity-based discrimination.

The Brazilian health system, regulated in 1990 and called the Unified Health System (SUS), is mostly composed of public and free health services, complemented by some services from the private network financed by the State^7^. In addition, SUS is constituted by doctrinal principles (universality, equity and integrality), to which the health care model, expressed in policies, programs, services organization and care provision, must turn to, in order to recognize the social determinants of the health-disease process and health inequalities^8^.

Based especially on the principle of equity, health care practices should be carried out in a more fruitful way to serve the most vulnerable people, including transvestites and transgender people. Initiatives aimed at this population have been implemented in the SUS over the years, such as the National LGBT Comprehensive Health Policy, created in 2011, and the Transsexual Process Program in the SUS, created in 2008 and expanded in 2013^9^.

In the scientific literature, there has been an increase in the publication of articles on the health of the Brazilian LGBTTQIA+ population after the creation of the National LGBT Integral Health Policy^10^, as well as theses and dissertations on transvestism, transsexuality and health, especially after the expansion of the Transsexualizing Process in the SUS^11^. Furthermore, it is possible to identify some integrative reviews on the health of the transvestite and transsexual population in Brazil, specifically on the difficulties these people face in accessing health services^12 , 13^. However, there are no systematic reviews that present summarized evidence on the other aspects related to health care for the Brazilian transvestite and transsexual population.

Considering that Brazil is a country of continental dimensions, with diverse and profound inequalities, especially in the realization of the universal right to health, the question is: how has health care for transvestites and transsexuals been provided within the scope of the SUS? As a result, the objective of this study is to synthesize scientific evidence to characterize health care for transvestites and transsexuals in the country.

## METHODS

This is a systematic review of the scientific literature, conducted from July 2020 to January 2021 and updated in September 2021. The research protocol^14^followed the recommendations of the checklist Preferred Reporting Items for Systematic Review and Meta-analysis Protocols (Prisma-P)^15^, and is registered on the International Prospective Register of Systematic Reviews (Prospero) platform, under code CRD42020188719.

From the formulation of a well-defined problem-question and an explicit and reproducible methodology, systematic review studies are able to identify, select, evaluate and summarize already available scientific evidence^16^. Likewise, they can point out necessary changes to professional practices or recommendations for carrying out other investigations, aiming to fill gaps in knowledge.

The survey of evidence, in Portuguese and English, that integrates this systematic review was carried out from consultations to the following databases: Scientific Electronic Library Online (SciELO), US National Library of Medicine (Pubmed), Literatura Latino-Americana e do Caribe em Ciências da Saúde (Lilacs) and Biblioteca Virtual em Saúde (BVS). The BVS, a digital platform coordinated by the Centro Latino-Americano e do Caribe de Informação em Ciências da Saúde (BIREME), gathers data from different electronic databases in the health area, such as: Base Regional de Informes de Avaliação de Tecnologias em Saúde das Américas (Brisa), Littérature Scientifique in Santé (Lissa), Medical Literature Analysis and Retrieval System Online (MedLine), Sistema de Información de la Biblioteca de la Organización Mundial de la Salud (Wholis), Índice Bibliográfico Español en Ciencias de la Salud (IBECS), Base de Dados em Enfermagem (BDENF), Bibliografia Brasileira de Odontologia (BBO), among others.

To conduct the electronic search of the studies, previously defined strategies were used after different attempts, consisting of a block of health care-related descriptors in the Brazilian context and a block of keywords related to transvestites and transgender people, respecting the specificities of each database ( Box 1 ).

**Box 1:** Search strategies for the selected databases. Natal, Brazil, 2022.

Database	Search strategy
SciELO	(“atenção à saúde” OR “sistema único de saúde” OR SUS OR brasil) AND (travesti OR travestilidade OR travestismo OR transexual OR transexualidade OR transexualismo OR transgênero OR “pessoas trans”)
PubMed	(“health care” OR “health systems”) AND brazil AND (“transgender persons” OR transvestism OR transsexualism)
Lilacs	(“atenção à saúde” OR “sistema único de saúde” OR SUS OR brasil) (travesti OR travestilidade OR travestismo OR transexual OR transexualidade OR transexualismo OR transgênero OR “pessoas trans”)
BVS	(“atenção à saúde” OR “sistema único de saúde” OR SUS OR brasil) (travesti* trans*)

In the SciELO, Lilacs and BVS databases, the search strategies were composed of descriptors extracted from the vocabulary of the Descritores em Ciências da Saúde (DECS) and, for the search in PubMed, equivalent terms from the Medical Subject Headings (MESH) were used. Strategies were applied covering all indexes (title, abstract, keywords and text).

Eligibility criteria were based on the PECOS anagram^16^, with the necessary adaptations ( Box 2 ).

**Box 2:** Research elements according to the PECOS anagram. Natal, Brazil, 2022.

Element	Abbreviation	Description
Participants	P	Transvestites, transgender women and men, health professionals, health managers.
Exposure	E	Health policies and programs, health services organization, health care provision.
Comparison or control	C	NA
Outcome	O	Health promotion, access to health services, responses to health demands and needs.
Types of study included	S	Original studies with a qualitative or quantitative approach.

NA: Not applicable.

Source: Authors’ preparation. Adapted from Galvão and Pereira ^16^ .

The adaptation in the PECOS anagram concerned the comparison or control element, not being considered for the inclusion criteria of this study. Therefore, articles resulting from original studies were included in the review, with a qualitative or quantitative approach, on aspects related to health care for the transvestite and transsexual population in Brazil, and in which the subjects were transvestites, transgender people, health professionals or health managers. Furthermore, the studies included were those published in full in scientific journals from 1990 onwards, the year of SUS regulation, regardless of language.

Studies that addressed health care for transvestites and transsexuals only in the private health system or together with aspects related to health care for other people in the LGBTTQIA+ group were excluded, as well as studies classified as abstracts published in annals, editorials, reflective articles, documentary analyses, literature reviews, technical manuals, chapters, books, monographs, dissertations and theses.

The publications were retrieved by a single researcher and subsequently coded and organized in an electronic spreadsheet for later removal of duplicates. Next, considering the inclusion and exclusion criteria adopted, screening was performed based on the reading of the titles and abstracts of the files, to select those that could be included in the systematic review and that would be read in full.

The full reading stage was carried out to elect those studies with strong potential for inclusion in the systematic review. Through manual search and in a complementary way, the reference lists of the selected articles were also consulted to identify possible misses during the electronic search.

The selected articles were read again in full so that their methodological quality could be assessed by two previously calibrated researchers, aiming to guarantee uniformity in the critical evaluation. This happened through the critical evaluation tools of the Joanna Briggs Institute (JBI), using a specific checklist for studies with a qualitative approach^17^, since all eligible articles had this methodological approach.

Based on the checklist presented by Lockwood et al.^17^, as well as the classification proposed by Almeida et al.^18^, the risk of bias in the articles was rated as high (with up to 49% of affirmative responses), moderate (affirmative responses between 50% and 69%) and low (70% or more of affirmative responses). In this review study, the final synthesis was composed only of articles with low risk of bias.

The entire process of screening, eligibility and assessment of the methodological quality of the articles was carried out by two independent researchers. In case of doubt or disagreement, the researchers met virtually to discuss and establish a consensus.

The synthesis of the findings was carried out through the formal narrative and by preparing tables containing some data of the articles: title, authorship, year of publication, objective, sample/target audience, place of study (Federative Unit of Brazil, i.e., state), type of study, methodological approach, results and conclusions.

The results of the studies included in the systematic review were grouped categorically, according to their thematic approaches, and interpreted with the support of the scientific literature that deals with the issue investigated here. Finally, the final writing of this manuscript followed the recommendations of the Preferred Reporting Items for Systematic Reviews and Meta-analyses checklist (Prisma)^19^.

## RESULTS

**Figure 1:**
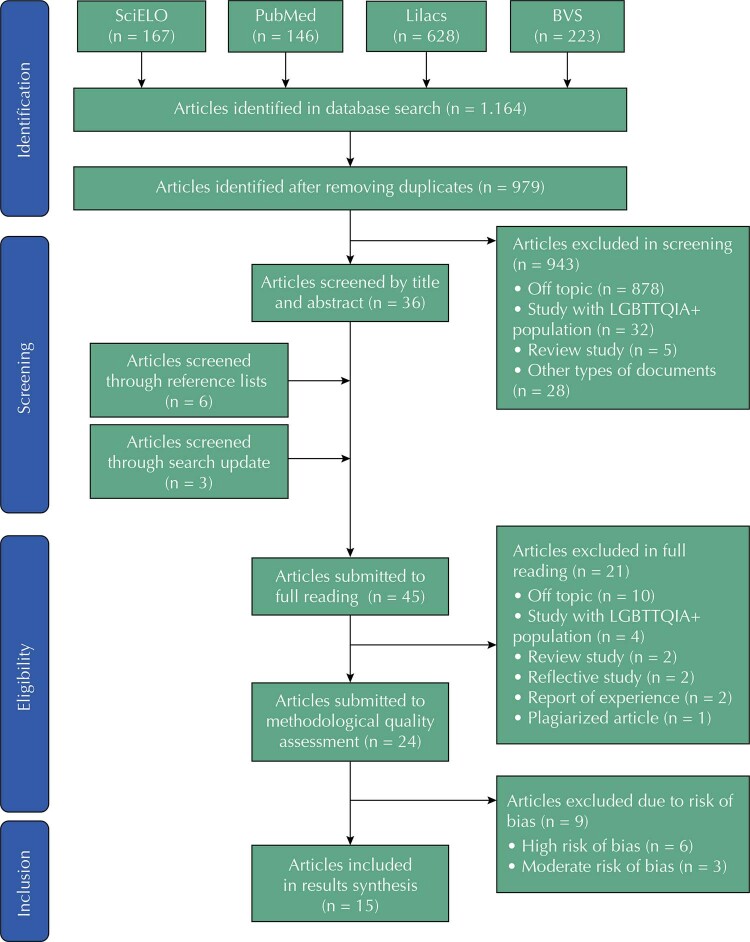
Flowchart of the process of identification, screening, eligibility and inclusion of articles in the systematic review. Natal, Brazil, 2022.

Source: Authors’ preparation. Adapted from Flor et al. ^20^ .

By searching the databases of interest for this systematic review, it was possible to retrieve 1,164 titles. After removing duplicates, screening after reading titles and abstracts, including other publications by checking the references of the screened articles, and updating the search, 45 articles were submitted to full reading. Of these, 24 were submitted to methodological quality assessment and 15 were included in the results synthesis, with 14 articles published in Portuguese and one article published in English ( Figure 1 ).

The 15 articles included in this systematic review do not meet the sixth criterion of the checklist used to guide the assessment of methodological quality^17^, that is, they do not present explicit information about the researchers’ cultural or theoretical location and its possible influence on the study ( Box 3 ).

**Box 3:** Assessment of the methodological quality of the articles included in the systematic review, according to the checklist criteria used 17 and the classification proposed by Almeida et al. ^18^ . Natal, Brazil, 2022.

Articles	Checklist evaluation criteria										Risk of bias (%)^b^

	1	2	3	4	5	6	7	8	9^a^	10	
Oliveira e Romanini ^21^	A	A	A	A	A	B	A	A	A	A	Low (90%)
Rigolon et al. ^22^	A	A	A	A	A	B	A	A	A	A	Low (90%)
Sousa e Iriart ^23^	A	A	A	A	A	B	C	A	A	A	Low (80%)
Moraes e Silva ^24^	A	A	A	A	A	B	B	A	A	A	Low (80%)
Sehnem et al. ^25^	B	A	A	A	A	B	B	A	A	A	Low (70%)
Amorim e Teixeira ^26^	C	A	A	A	A	B	B	A	A	A	Low (70%)
Sehnem et al. ^27^	B	A	A	A	A	B	B	A	A	A	Low (70%)
Almeida et al. ^28^	B	A	A	A	A	B	B	A	A	A	Low (70%)
Souza et al. ^29^	C	A	A	A	A	B	B	A	A	A	Low (70%)
Oliveira et al. ^30^	B	A	A	A	A	B	B	A	A	A	Low (70%)
Sevelius et al. ^31^	A	A	A	A	A	B	C	A	C	A	Low (70%)
Lovison et al. ^32^	B	A	A	A	A	B	B	A	A	A	Low (70%)
Ferreira et al. ^33^	C	A	A	A	A	B	B	A	A	A	Low (70%)
Hanauer e Hemmi ^34^	A	A	A	A	A	B	C	A	C	A	Low (70%)
Silva et al. ^35^	B	A	A	A	A	B	B	A	A	A	Low (70%)

Legend: A = yes, B = no, C = not clear.

^a ^To meet the criteria, the inclusion of the number of the ethical approval opinion in the text of the article was considered.

^b ^Percentage of affirmative answers.

Source: Authors’ preparation. Adapted from Flor et al. ^20^

In studies with qualitative approaches, researchers’ beliefs and values, as well as their theoretical orientations, can influence the conduct of the research, from data collection to results dissemination. Therefore, it is important that articles that value methodological rigor explain the possible influences of the researchers in the study, or the strategies adopted to reduce these potential influences^17^.

The synthesis of the included studies ( Box 4 ) reveals that 10 articles, i.e., the majority, were published between 2017 and 2019. The research that originated these articles was developed in the South and Southeast of Brazil, five of them in each region. Therefore, articles resulting from studies carried out in the North region were not included.

**Box 4:** Summary of the main characteristics of the articles included in the systematic review. Natal, Brazil, 2022.

Authors	Year	State	Research subjects	Aim	Results	Conclusions
Oliveira e Romanini ^21^	2020	RS	6 transgender women.	To understand how the trajectories of transgender people are constructed in public health policies in a municipality in the interior of Rio Grande do Sul.	Trans women have a closer relationship with the transsexualizing outpatient clinic. However, a fragile relationship is built in this environment, as users feel constantly evaluated regarding the “truthfulness” of their transsexuality, becoming passive in the health production process due to the superiority of medical knowledge and reproduction of invisible protocols.	The journey of trans women in public health policies is guided by invisibility, leading to the construction of lines of care that do not match the real needs of this population group. It is necessary to encourage the teaching-service integration, aiming to build a dialogue between scientific knowledge about transsexuality and the experiences of trans people, to qualify health workers and, consecutively, the health care of trans women.
Rigolon et al. ^22^	2020	SP	1 transvestite, 1 transgender woman and 2 transgender men.	To understand the life stories and journey of transvestites and transsexuals in health services.	The results were presented under two main themes: “gender and sexuality in life histories” and “trajectories in health services”. The reports reveal the challenges faced by trans people in the process of recognizing their gender identity and the dilemmas in accessing health services, keeping them away from care spaces.	The Oral History method can increase knowledge about the health needs and demands of transvestites and transsexuals. In addition, the research results can help health professionals and managers in decision-making and comprehensive care for these people.
Sousa e Iriart ^23^	2018	BA	10 transgender men.	To understand the health needs and demands of trans men, considering this to be a first step towards enabling ways in which care strategies can be designed.	Transphobia, a structural and institutional mechanism of violence and aversion to trans people, determines care practices and the development of strategies to meet the health needs and demands of trans men. These, in turn, are presented in three categories: “de-pathologization of trans experiences”, “body modifications” and “outpatient care”.	Transphobia negatively influences the way of life of trans men, aggravating their health needs and demands. It is necessary to develop intersectoral strategies to combat transphobia and guarantee the right to health and life of trans men.
Moraes e Silva ^24^	2020	RN	7 transvestites.	To identify conceptions, desires, fears and suggestions regarding humanized health care aimed at transvestite people in the context of primary care.	Transvestites face difficulties in accessing and using health services. To overcome them and change the health care of transvestites, some suggestions are pointed out: training of health professionals, dialogue with the social movement, publicity campaigns for getting closer together with the meaning of the experience of being a transvestite.	For primary health care to be a welcoming and humanized space for transvestites, it is necessary to prioritize respect and guarantee of rights. It is necessary to rescue the promotion of care, something fundamental in health work practices, respecting differences and exercising altruism. There is an urgent need for effective changes in the training of health professionals.
Sehnem et al. ^25^	2017	RS	10 nurses.	To get to know health care for transvestites, from the perspective of nurses, in primary care.	Two thematic categories emerged: “weaknesses in meeting the demands of transvestites in primary health care” and “welcoming as an integrating practice in primary health care”. Nurses do not feel prepared to welcome transvestites and believe that they do not seek Basic Health Units for fear of being embarrassed.	The few health actions aimed at transvestites occurred in an isolated and fragmented way. To overcome the challenges, it is recommended the implementation of the LGBT health policy in the SUS, the permanent education of health workers and the strengthening of reception to enhance the bond between professionals and transvestite users.
Amorim e Teixeira ^26^	2017	RJ	5 transvestites.	To discuss meeting transvestites’ health needs in primary health care services.	For the interviewees, the health condition is linked to health promotion actions. In the search for body modifications, transvestites make use of female hormones without monitoring, leading to the emergence of other demands, such as cardiovascular complications. When seeking the Basic Health Units (UBS), transvestites face disrespect for their social name and other violations, which distance them from these health services, bringing them closer to alternative or clandestine practices for self-care.	Primary care is still fragile to meet transvestites’ health needs and demands. It is recommended to implement a public health policy that considers transvestites’ specific health issues to promote comprehensive health care; include content on the care of transvestites in health area courses and develop permanent educational actions, on reception and humanization, for health professionals working in primary care.
Sehnem et al. ^27^	2017	RS	10 nurses.	To know the nurses’ perceptions about transvestites and the technical-scientific preparation to assist them.	Data were presented in two thematic categories: “transvestite: meanings constructed from the perspective of nurses” and “(lack of) technical-scientific preparation: repercussions on the care provided to transvestites”. Nurses are unaware of the meaning of being a transvestite and public policies aimed at the LGBT population, one of the reasons given for this lack of knowledge being the fragile approach to gender and sexuality issues during academic training.	For the care of transvestites, it is necessary to rethink public health policies for this part of the population, guarantee access and reception in health services, in addition to qualifying the technical-scientific preparation of health professionals on gender and sexuality issues.
Almeida et al. ^28^	2018	RJ	13 nurses.	To identify the training of resident nurses for the qualified care of transgender people and to analyze the process of nursing care for this clientele, from the perspective of the nursing resident.	The testimonies were presented in two categories: “nurse education and care for transgender people” and “the process of nursing care from the resident’s perspective”. In the residents’ view, the training of nursing professionals is not aware of the particularities of different gender identities, including those of transgender people. Thus, the unpreparedness of these professionals to provide qualified care, given the needs of transgender people, will be perpetuated.	The unpreparedness of nursing professionals to meet the social and health demands of the transsexual population was evidenced, justified, above all, by the absence of curricular content on transsexuality during professional training.
Souza et al. ^29^	2014	RS	49 transvestites.	To present the therapeutic itineraries of transvestites residing in Santa Maria, a municipality in the central region of Rio Grande do Sul.	It was found that transvestites seek multiple ways to obtain health care, including outside institutionalized health services. For a better understanding of the transvestites’ therapeutic itineraries, the results of the study were presented in four categories: “the trajectory of the transvestite construction”, “the itinerary of care in the spaces covered by transvestites”, “the itinerary in public health services” and “the itinerary in the Afro religion, the *batuque* ”.	The study revealed that transvestites avoid public health services, because when they seek them they are always victims of discrimination. Such health services are not prepared to meet their needs and demands. Therefore, the therapeutic itineraries of the transvestites in Santa Maria, Rio Grande do Sul, are constituted by other scenarios and health practices, in addition to the institutional spaces of the SUS, such as living spaces, prostitution points, in public spaces and religious meeting places ( *casas de santo* ).
Oliveira et al. ^30^	2019	DF	2 transgender people.	To understand how trans social movements in the Federal District understand the right to health.	It is understood that the right to health is related to three thematic units: “right to exist”, such as the search for basic human rights (use and respect for the social name); “right to equity”, since health services must be prepared to meet the specific needs of the trans population; and “implementation of the trans clinic and social participation”, highlighting the importance of social control for the realization of the right to health.	The fight for recognition of the specific needs of the trans population and the winning of social equipment to satisfactorily serve this population, such as the trans outpatient clinic, are initiatives that can provide a permanent dialogue between trans people and health professionals for the production of specific and qualified care, reverberating in the realization of the right to health and citizenship.
Sevelius et al. ^31^	2019	RJ	36 transgender women.	To explore how the social context of stigma and transphobia affect Brazilian trans women’s access and their preferences for HIV prevention method and care programming guided by the Gender Affirmation Model.	The results portray discrimination in health services, from disrespecting the social name to the prior conception that these users are people living with HIV. Transgender women are uncertain about the effectiveness and delivery of Pre-Exposure Prophylaxis (PrEP), and are not emotionally prepared to face a possible positive HIV result. Group activities can explore other issues, in addition to HIV, ensuring the possibility of coexistence between seropositive and non-seropositive people. The interviewees demonstrate a feeling of confidence when faced with health services that have transsexual workers in the team.	The data strongly support a gender-affirming approach to transgender health, in which HIV programming for transgender women is not limited to individual and biomedical strategies such as increased PrEP intake or increased testing rates. There is a need to affirm the diverse subjectivities of transgender women and support their autonomy as they cultivate health and empowerment, both for themselves and their communities.
Lovison et al. ^32^	2019	SC	4 transgender women and 1 transvestite.	To know the perception of transvestites and transsexuals living in Chapecó, Santa Catarina, regarding access and health care.	The search for care in public health services is not a priority for the interviewees. They end up opting for self-medication or paying the costs of private assistance. This stems from the discriminatory acts they face in these services, mainly disrespect for the social name and lack of knowledge on the part of health professionals about health policies and programs for this specific population.	Access and health care are limited and fragmented due to the small number of professionals and health services that meet the general demands of the population of the municipality of Chapecó, and the lack of preparation of the municipal health system to meet the specific demands of the trans population. Trans women do not have basic rights met, such as the right to health; they do not receive information about health processes and procedures and are discriminated against by health professionals and other users within health services.
Ferreira et al. ^33^	2017	IP	6 transvestites.	To analyze and understand transvestites’ experiences regarding health care in the SUS in Teresina, Piauí.	Two thematic categories emerged: “weaknesses in transvestite care” and “specialization in transvestite care “. The first category reveals situations of prejudice in health services due to body changes, generating insecurity on the part of this population when using services. The second category suggests that the creation of specific services for transvestites could minimize situations of embarrassment.	It was evident that there is a need for greater integration between the different social segments and health services, the qualification of professionals to guarantee the reception of transvestites and urgent reflection on the discriminatory nature of the specialized services implemented by SUS, even though it is a door for transvestites to enter the health system.
Hanauer e Hemmi ^34^	2019	MG	4 transgender men and 3 transgender women.	To describe the paths traversed by transsexuals, aiming to know their itineraries in the search for meeting their health needs and demands.	The different paths presented are related to the process of identifying as a transsexual person and the influence of social networks, as well as the health services in this path. Health care networks were recognized as central in the lives of trans people interviewed, especially with regard to access to body modifications.	The search for the desired gender identity is crossed by numerous difficulties, such as family and social non-acceptance and denial of the right to health when seeking SUS services. Transsexuality seems to be misunderstood by people in social life and by professionals and managers of health care networks.
Silva et al. ^35^	2014	RN	12 nurses.	To analyze the practice and knowledge of nurses from the Family Health Strategy (ESF) regarding care to the transvestite population.	The results of the study are presented from three discursive axes. The first axis concerns the construction of the transvestite identity, how the interviewed professionals understand this gender identity and how they develop nursing practices for this population group. In the second axis, the nurses reveal that they have not treated transvestites in primary care or that they do not know how to identify them. The third axis presents initiatives by some professionals to better welcome and assist transvestites, such as respect for the social name.	Nurses demonstrated that practice and knowledge in assisting transvestites are limited, failing to identify them by gender of choice, judging them only by their physical and apparent characteristics. Due to this invisibility, they only perform a curative service, without considering the transvestites’ subjectivities and respect for the social name, disqualifying the care for this specific public.

In their entirety, the articles have a qualitative approach as the only methodological characteristic. In addition, six of the articles had transvestites and transgender women as research subjects and three were developed with health professionals, specifically nurses, focusing on their perception of health care for transvestites and transsexuals.

According to the summary contained in Box 4 , eight articles (the majority) have as their focus of analysis the access of transvestites and transsexuals to health services and the care offered to this public. From the thematic approaches identified in the results of the articles, the findings of this review were grouped into six categories, presented below.

### Possibilities to Transform Health Care

With regard to health care for transvestites and transsexuals in Brazil, the articles reveal, almost unanimously, the existing difficulties for implementing the basic principles of the SUS. In view of the diagnosis of these difficulties, which will be addressed in the following categories, possibilities are pointed out to transform this health care model, such as: development of intersectoral strategies to combat discrimination against transvestites and transsexuals, including in health services^23 , 33^; constant dialogue between SUS management and the social segments that represent transvestites and transgender people, with a view to understanding and respecting the health specificities of this part of the population^24 - 26 , 30 , 33^; implementation of the National LGBT Comprehensive Health Policy, guaranteeing access for transvestites and transsexuals to the different establishments of the SUS^25 - 27^; strategies to disseminate the meaning of being a transvestite or transsexual to the community in general, aiming to assert subjectivities and respect these people’s needs^24 , 31^; review of work practices in health, involving the rescue of the promotion of care, altruism by health professionals, development of autonomy for transvestites and transsexuals and inclusion of transsexual workers in health teams^24 , 25 , 31^; and changes in professional training in health, encouraging the integration of teaching-service-community, the inclusion of content on gender and sexuality in the curricula of courses in the health area and the development of permanent education actions regarding humanized care for transvestites and transsexuals to SUS workers^21 , 24^.

### Transvestiphobia and Transphobia: Violations Inside and Outside the SUS

In the course of their lives, transvestites as well as transgender women and men are victims of prejudiced attitudes, a reflection of structural violence (transvestiphobia and transphobia) caused by the simple fact that it is a group that breaks with the hegemonic pattern, which tries to define the gender only by the anatomy of genital organs.

This aversion soon emerges within the family, when transvestites and transsexuals in search of recognition of their gender identities are not accepted by the family itself. This non-acceptance also occurs on the part of people from social circles^22 , 34^. In addition, more than structural, transvestitephobia and transphobia are also institutional violence, that is, they are present in institutions such as the SUS.

Therefore, recognizing the violence suffered by transvestites and transsexuals must be part of the work process in the health area, in order to try to welcome and support the victims. However, discriminatory acts, such as disrespect for the social name and lack of understanding about transvestism and transsexuality on the part of health professionals and managers^31 , 34^, negatively influence health care practices for this specific public.

As a result, transvestites and transsexuals feel insecure when using health services^33 , 34^, even avoiding them^22 , 29^. In the case of transsexual men, as reported by Sousa and Iriart^23^based on a study carried out in Salvador, Bahia, institutional transphobia prevents their access to health services that meet their specific needs and demands, including hormonal monitoring for body modifications.

Data from a study with Brazilian transsexual women reveal that health professionals’ previous conception that they would live with the Human Immunodeficiency Virus (HIV), among other Sexually Transmitted Infections (STIs), causes these users to stop seeking SUS services, considered discriminatory by these women^31^. In addition, the prejudice and stigma of health professionals related to them ends up keeping them away from access to combined HIV prevention strategies, such as the use of PrEP^31^.

### Professional Unpreparedness to Care for Transvestites and Transsexuals

The findings reveal that health professionals, especially nursing professionals, are not properly qualified to welcome and care for transvestites and transgender people in health services^25 , 27 , 28 , 35^. In their view, the unpreparedness for health care for this specific public is due to the fragile approach to questions about gender diversity and sexuality during the professional training process^27^.

As a result, some nurses are unaware of the subjectivities of a transvestite or transsexual person and their health needs and demands^27 , 28^. In addition, the absence of permanent educational actions, which disseminate the guidelines of the National Policy for Integral LGBT Health and which qualify the care for transvestites and transsexuals in the SUS, makes nursing professionals perpetuate stigmas and continue to limit care for these people, disrespecting them and basing their care practices only on curative actions^35^.

### Search for Health Care Alternatives

Due to professional unpreparedness, and the discriminatory acts experienced within SUS establishments already pointed out in the results of this article, transvestites and transsexuals avoid seeking care in these institutional spaces^22 , 29 , 32 - 34^. Faced with these violations, a minority of transvestites and transsexuals opt for private assistance, but most cannot afford a health plan, becoming dependent on alternative self-care practices, on the support of people in the social network or religious spaces of African origin, or body transformations without proper professional follow-up^26 , 29 , 32^.

In this context, body transformations, such as inappropriate hormone therapy, industrial silicone grafting and breast self-mutilation, can cause other health problems, such as cardiovascular and aesthetic complications. This clearly characterizes another risk factor for the lives of transvestites and transgender people^26^.

### Right to Health for Transvestites and Transsexuals: Utopia or Reality?

According to the Federal Constitution of Brazil, access to health is a basic right for all people, guaranteed through public policies aimed at reducing the risk of diseases and injuries, as well as allowing universal and equal access to health actions and services^36^. However, for Brazilian social minorities, such as transvestites and transsexuals, this fundamental right has not been real.

The right to health for transvestites and transsexuals, or the lack of it, can be understood by three aspects: the right to exist, considering that these people still struggle to have their social names respected, including in the SUS; right to equity, as health services are not yet prepared to assist transvestites and transsexuals; and social participation, because transvestites and transsexuals need to exercise social control to access and remain in health services, and to demand the creation of specific services to offer transsexualizing procedures^30 , 32 , 34^.

### The Transsexualization Process: Advances and Challenges

In Brazil, the transgender procedures, on an experimental basis, have been authorized by the Federal Council of Medicine (CFM) since 1997, being incorporated into the SUS in 2008, when the Ministry of Health founded the Transsexualizing Process program and formally recognized that body transformations are also health needs of the transvestite and transsexual population^9^. Such procedures, as well as the creation of qualified services to offer them, represent a step forward in promoting the health of transvestites and transsexuals.

Therefore, it is expected that the services enabled to offer the procedures provided for in the Transsexualization Process are welcoming, discrimination-free environments, as they are made up of multidisciplinary teams duly qualified to care for transvestites and transsexuals in a humane way^33^. However, there is a fragile bond between health professionals and users, especially with trans women^21^.

According to Oliveira and Romanini^21^, this situation can be explained by the existence of medical superiority and a care protocol, lasting two years, which treats gender identity transition as a disorder, making this diagnosis a condition for access to specific health services for this part of the population. As a result, in these services, transvestites and transsexuals are constantly asked about the veracity of their gender identities and intentions, not allowing them autonomy in the production of care.

## DISCUSSION

The health care model consists of a set of knowledge and combinations of resources (financial, technological, human, etc.) to meet individual and collective health needs^37^. Therefore, analyzing the health care model, whether for the general population or for specific groups, such as the group of transvestites and transgender people, implies understanding what public health policies exist to mitigate or nullify inequities and how these policies reflected in programs, actions, services and research for this purpose^38^.

In view of the results of this systematic review, it is clear that health care for transvestites and transsexuals in Brazil is still exclusionary, fragmented, centered on specialized care and guided by curative actions, resembling the care models that preceded the SUS and which have been strongly criticized since the 1970s, from the Brazilian Sanitary Reform movement. At the time, the critics highlighted the importance of profound changes not only in the health system, so that care was humanized, equitable and comprehensive, guaranteeing universal access to all men and women^38^.

According to Arán et al.^39^, despite transsexuality being a phenomenon recognized since the late 19th century, issues related to transgender identities in the Brazilian public health began to be visible only after 1979, with the possibility of medical interventions, when the CFM was consulted for the first time on mammoplasty in transgender people.

Going forward in time, and acknowledging the existence of numerous debates that took place on the subject, including in the legal field, in 1997 – as already discussed in this article – the reassignment procedures were authorized by the CFM and incorporated into the SUS 11 years later^9^. It should be noted that the existence of these procedures in the SUS is a fundamental initiative not only to meet a need for transvestites and transsexuals, but also to assist in the construction of subjectivities and identities neglected by society and the State.

However, criticisms are made of the care protocols established by the Ministry of Health, through regulations^40 , 41^, so that transvestites and transsexuals can benefit from these specialized health services, especially psychiatric follow-up for at least two years in order to confirm the diagnosis of transsexuality. While this diagnosis represents the winning of the right to health for transvestites and transsexuals, it also contributes to the permanence of the stigma on these people, as it does not consider the personal and historical issues of each subject, assigning the recognition of gender identity to a psychiatric and normalizing procedure^39^.

Furthermore, access to reassignment procedures is not the only health need for transvestites and transsexuals in Brazil. In addition to access to these procedures, it is necessary to promote the fight against discrimination based on gender identity so that transvestites and transsexuals can access any space with dignity, such as health promotion spaces, without being victims of violence^26^.

If access to gender reassignment procedures were the only health need of Brazilian transvestites and transsexuals, it would be very unequal and far from being met, since in the country there are only ten services authorized by the Ministry of Health to offer the procedures provided for in the Transsexualizing Process program, most of them located in the Southeast and none in the North^9^.

The findings of this systematic review also reveal possible ways to transform the current health care model for transvestites and transsexuals in the SUS: it would be interesting to develop intersectoral strategies to combat discrimination against transvestites and transsexuals^23 , 33^, constant dialogue between SUS management and the social segments that represent transvestites and transsexual people ^24 - 26 , 30 , 33^, the implementation of the National LGBT Comprehensive Health Policy^25^, strategies to disseminate the meaning of being a transvestite or transsexual to the community in general^24 , 31^, review of work practices in health^24 , 25 , 31^and changes in professional training in health^21 , 24^.

Considering these possibilities, the relevance of the participation of social movements representing this segment in the formulation and conduction of public health policies, through the exercise of citizenship and social control, is highlighted here. In the process of building the SUS and its health policies, joining the social struggle for the country’s re-democratization, the LGBTTQIA+ movement played an important role, placing sexual and gender diversity on the agenda as social markers and structural determinants of the health-disease process^42 , 43^.

As a result of the work of LGBTTQIA+ social movements in health, we highlight the creation of the Brazil without Homophobia Program and the Technical Committee on Health of the LGBT Population at the Ministry of Health in 2004, the holding of the 1st National Seminar on Health of the LGBT Population in 2007, the institution of the Transsexualizing Process in the SUS in 2008, the regulation of the use of the social name of transvestites and transsexuals in the SUS in 2009, the formulation of the National Policy for Integral LGBT Health, etc.^44^

In conclusion, health care for transvestites and transsexuals in Brazil does not correspond to the health care model advocated in the legal-legal framework of SUS. Therefore, it is necessary to develop strategies to deal with specific issues of transvestites and transsexuals, with emphasis on the vulnerabilities that permeate these people’s lives.

In addition to guaranteeing health care, it is necessary to provide opportunities for access to education, employment, housing, food, etc. These actions should not only come from transvestites and transsexuals, but from those who believe in the potential of the SUS and in an egalitarian and democratic society.

With regard to the actions of health professionals in relation to transvestites and transsexuals, this analysis focused on nursing professionals, as the studies retrieved and included in this review addressed only this professional category. In order not to induce that the professional lack of preparation for the health care of transvestites and transgender people in the Brazilian health system is a specificity of nursing professionals, and considering that the work in the SUS must be interprofessional and collaborative, further studies are recommended that investigate the way in which other health professionals deal with transvestites and transgender people.

At the same time that criticisms are made, it is important to highlight that this analysis about the work of nurses, in favor of care for transvestites and transsexuals, may result from advances in research on gender diversity in the field of nursing. However, as shown in the results presented here, it is still necessary to change work practices, allowing a significant transformation in the daily life of health care spaces for transvestites and transsexuals.

As limitations of the study, we point out the possibility that some articles do not use the descriptors adopted in the search strategies of this review and the existence of other articles indexed in databases not consulted. However, to bypass this possible situation, manual searches were performed.

In addition, other limitations refer to the non-inclusion of studies developed in the North region of Brazil, considering that the SUS is present throughout the national territory, and to the authors’ choice to exclude from the systematic review articles that analyzed health care for transvestites and transsexuals together with aspects related to health care for other people in the LGBTTQIA+ group.

The non-inclusion of articles that deal with health care for transvestites and transsexuals in states in the North region is justified by the fact that existing studies did not meet the inclusion criteria adopted in the systematic review, or did not have a low risk of bias, according to the checklist used to assess the methodological quality of the articles^17^.

The choice to exclude articles that addressed health care for transvestites and transsexuals together with health care for other people in the LGBTTQIA+ group is justified by virtue of the National Policy for LGBT Integral Health, which partially recognizes the specific needs and identities of transvestites and transsexuals, because, despite the existence of intersections between gender and sexuality issues, they demand different analyses and investments.

In this sense, it would be important to put in place new policies and specific programs for the health of transvestites and transsexuals, prioritizing coping with the vulnerabilities faced by these people^11 , 45^. However, these decisions may have limited the results of the systematic review, as it is expected that aspects about transvestites and transsexuals will be addressed together with those related to lesbian, gay, bisexual, non-binary people, etc., due to the existence of a single health policy for the entire LGBTTQIA+ population in Brazil.

## References

[1] Jesus JG (2012). Orientações sobre identidade de gênero: conceitos e termos: guia técnico sobre pessoas transexuais, travestis e demais transgêneros, para formadores de opinião.

[2] Ferreira ACG, Coelho LE, Jalil EM, Luz PM, Friedman RK, Guimarães MRC (2019). Transcendendo: a cohort study of HIV-infected and uninfected Ttransgender women in Rio de Janeiro, Brazil. Transgend Health.

[3] Reisner SL, Poteat T, Keatley J, Cabral M, Mothopeng T, Dunham E (2016). Global health burden and needs of transgender populations: a review. Lancet.

[4] Narang P, Sarai SK, Aldrin S, Lippman S (2018). Suicide among transgender and gender-nonconforming people. Prim Care Companion CNS Disord.

[5] Mendes WG, Silva CMFP (2020). Homicídios da população de lésbicas, gays, bissexuais, travestis, transexuais ou transgêneros (LGBT) no Brasil: uma análise espacial. Cien Saude Colet.

[6] Mendes WG, Duarte MJO, Andrade CAF, Silva CMFP (2021). Revisão sistemática das características dos homicídios contra a população LGBT. Cien Saude Colet.

[7] Paim JS (2018). Sistema Único de Saúde (SUS) aos 30 anos. Cien Saude Colet.

[8] Fertonani HP, Pires DEP, Biff D, Scherer MDA (2015). Modelo assistencial em saúde: conceitos e desafios para a atenção básica brasileira. Cien Saude Colet.

[9] Rocon PC, Sodré F, Rodrigues A, Barros MEB, Wandekoken KD (2019). Desafios enfrentados por pessoas trans para acessar o processo transexualizador do Sistema Único de Saúde. Interface (Botucatu).

[10] Prado EAJ, Sousa MF (2017). Políticas públicas e a saúde da população LGBT: uma revisão integrativa. Tempus Actas Saude Colet.

[11] Lima RRT, Flor TBM, Araújo PH, Noro LRA (2020). Análise bibliométrica de teses e dissertações brasileiras sobre travestilidade, transexualidade e saúde. Trab Educ Saude.

[12] Ferreira BO, Bonan C (2020). Abrindo os armários do acesso e da qualidade: uma revisão integrativa sobre assistência à saúde das populações LGBTT. Cien Saude Colet.

[13] Rocon PC, Wandekoken KD, Barros MEB, Duarte MJO, Sodré F (2020). Acesso à saúde pela população trans no Brasil: nas entrelinhas da revisão integrativa. Trab Educ Saude.

[14] Lima RRT, Flor TBM, Silva AB, Noro LRA (2020). Health care for transgender people in Brazil: a systematic review protocol. Res Sq [Preprint].

[15] Moher D, Shamseer L, Clarke M, Ghersi D, Liberati A, Petticrew M (2015). Preferred reporting items for systematic review and meta-analysis protocols (PRISMA-P) 2015 statement. Syst Rev.

[16] Galvão TF, Pereira MG (2014). Revisões sistemáticas da literatura: passos para sua elaboração. Epidemiol Serv Saude.

[17] Lockwood C, Munn Z, Porritt K (2015). Qualitative research synthesis: methodological guidance for systematic reviewers utilizing meta-aggregation. Int J Evid Based Healthc.

[18] Almeida GM, Oliveira KHD, Monteiro JS, Medeiros MAT, Recine EGIG (2018). Educational training of nutritionists in public health nutrition: a systematic review. Rev Nutr.

[19] Moher D, Liberati A, Tetzlaff J, Altman DG, PRISMA Group (2009). Preferred reporting items for systematic reviews and meta-analyses: The PRISMA statement. PLoS Med.

[20] Flor TBM, Cirilo ET, Lima RRT, Sette-de-Souza PH, Noro LRA (2022). Formação na Residência Multiprofissional em Atenção Básica: revisão sistemática da literatura. Cien Saude Colet.

[21] Oliveira I, Romanini M (2020). (Re)escrevendo roteiros (in)visíveis: a trajetória de mulheres transgênero nas políticas públicas de saúde. Saude Soc.

[22] Rigolon M, Carlos DM, Oliveira WA, Salim NJ (2020). “A saúde não discute corpos trans”: história oral de transexuais e travestis. Rev Bras Enferm.

[23] Sousa D, Iriart J (2018). “Viver dignamente”: necessidades e demandas de saúde de homens trans em Salvador, Bahia, Brasil. Cad Saude Publica.

[24] Moraes AND, Silva GSN (2020). Travestis e o cuidado humanizado em saúde. Rev Abordagem Gestalt.

[25] Sehnem GD, Rodrigues RL, Lipinski JM, Vasquez MED, Schmidt A (2017). Health Care Assistance in Primary Care: access to care. Rev Enferm UFPE On Line.

[26] Amorim JF, Teixeira ER (2017). Atendimento das necessidades em saúde das travestis na atenção primária. Rev Baiana Saude Publica.

[27] Sehnem GD, Rodrigues RL, Lipinski JM, Vasquez MED, Schmidt A (2017). (Des)preparo técnico-científico para o cuidado às travestis: percepções de enfermeiras(os). Rev Enferm UFSM.

[28] Almeida JSM, Martins ERC, Costa CMA, Moraes PC, Ferreira GDF, Spindola T (2018). Cuidar de pessoas transexuais na ótica dos residentes de enfermagem. Rev Enferm UERJ.

[29] Souza MHT, Signorelli MC, Coviello DM, Pereira PPG (2014). Itinerários terapêuticos de travestis da região central do Rio Grande do Sul, Brasil. Cien Saude Colet.

[30] Oliveira BP, Silva MAS, Souza MS (2019). O direito à saúde de pessoas trans* no Distrito Federal: entre o direito de existir e o direito à equidade. Cad Ibero Am Dir Sanit.

[31] Sevelius J, Murray LR, Fernandes NM, Veras MA, Grinsztejn B, Lippman SA (2019). Optimising HIV programming for transgender women in Brazil. Cult Health Sex.

[32] Lovison R, Ascari TM, Zocche DAA, Durand MK, Ascari RA (2019). Travestis e transexuais: despindo as percepções acerca do acesso e assistência em saúde. Enferm Foco.

[33] Ferreira BO, Nascimento EF, Pedrosa JIS, Monte LMI (2017). Vivências de travestis no acesso ao SUS. Physis.

[34] Hanauer OFD, Hemmi APA (2019). Caminhos percorridos por transexuais: em busca pela transição de gênero. Saude Debate.

[35] Silva GWS, Sena RCF, Santos QG, Sobreira MVS, Miranda FAN (2014). O dito e o feito: o enfermeiro e o saber/fazer saúde para travestis. Rev Enferm UFPE On Line.

[36] Brasil (1988). Constituição da República Federativa do Brasil.

[37] Gil CRR, Maeda ST, Soares CB, Campos CMS (2013). Fundamentos de Saúde Coletiva e o Cuidado de Enfermagem.

[38] Paim JS, Giovanella L, Escorel S, Lobato LVC, Noronha JC, Carvalho AI (2017). Políticas e Sistema de Saúde no Brasil.

[39] Arán M, Murta D, Lionço T (2009). Transexualidade e saúde pública no Brasil. Cien Saude Colet.

[40] Ministério da Saúde (BR), Secretaria de Atenção à Saúde (2008). Portaria Nº 457, de 19 de agosto de 2008. Aprova a regulamentação do Processo Transexualizador no âmbito do Sistema Único de Saúde (SUS).

[41] Ministério da Saúde (BR) (2013). Portaria Nº 2.803, de 19 de novembro de 2013. Redefine e amplia o Processo Transexualizador no Sistema Único de Saúde (SUS).

[42] Macrae E (2018). A construção da igualdade: política e identidade homossexual no Brasil da “abertura”.

[43] Galvão ALM, Oliveira E, Germani ACCG, Luiz OC (2021). Determinantes estruturais da saúde, raça, gênero e classe social: uma revisão de escopo. Saude Soc.

[44] Sena AGN, Souto KMB (2017). Avanços e desafios na implementação da Política Nacional de Saúde Integral LGBT. Tempus Actas Saude Colet.

[45] Bagagli BP, Oliveira AD, Pinto CRB (2017). Transpolíticas públicas.

