# Supplementary material for: Systematic review on health care for transvestites and transsexuals in Brazil
Source: Rev Saude Publica. 2023 Mar 29;57:19. doi: 10.11606/s1518-8787.2023057004693 (PMC10118414; doi:10.11606/s1518-8787.2023057004693)
Supplement: Translation

# Translation: Revisão sistemática sobre a atenção à saúde para travestis e transexuais no Brasil

**Authors:** Rafael Rodolfo Tomaz de Lima, Taiana Brito Menêzes Flor, Luiz Roberto Augusto Noro

**Affiliations:** I Brasil Universidade Federal do Rio Grande do Norte, Centro de Ciências da Saúde, Programa de Pós-Graduação em Saúde Coletiva. Natal, RN, Brasil

**Keywords:** Travestilidade, Transexualidade, Saúde das Minorias, Sistema Único de Saúde, Revisão Sistemática

## Abstract

**OBJETIVO:**

Sintetizar evidências científicas para caracterizar a atenção à saúde para travestis e transexuais no Brasil.

**MÉTODOS:**

Trata-se de uma revisão sistemática, conduzida de julho de 2020 a janeiro de 2021 e atualizada em setembro de 2021, cujo protocolo está registrado na plataforma *International Prospective Register of Systematic Reviews* (PROSPERO), sob o código CRD42020188719. O levantamento das evidências foi realizado em quatro bases de dados e os artigos elegíveis foram avaliados perante a qualidade metodológica, sendo incluídos aqueles com baixo risco de viés.

**RESULTADOS:**

15 artigos foram selecionados e os achados, de acordo com as suas aproximações temáticas, foram agrupados em seis categorias: Possibilidades para transformar a atenção à saúde; Travestifobia e transfobia: violações dentro e fora do Sistema Único de Saúde (SUS); Despreparo profissional para a atenção às travestis e transexuais; Busca por alternativas para a atenção à saúde; Direito à saúde para travestis e transexuais: utopia ou realidade?; O Processo Transexualizador: avanços e desafios.

**CONCLUSÕES:**

As evidências revelam que a atenção à saúde para travestis e transexuais no Brasil ainda é excludente, fragmentada, centralizada no cuidado especializado e pautada por ações curativas, assemelhando-se aos modelos de atenção que antecedem o SUS e que são fortemente criticados desde a Reforma Sanitária Brasileira.

## INTRODUÇÃO

As identidades transgêneras são diversas e não se limitam a uma definição específica. Porém, neste estudo, as pessoas transexuais são compreendidas como aquelas que possuem uma identidade de gênero oposta ao sexo atribuído ao nascimento^1^. Além disso, a denominação travesti antecede o termo transexual e é mais frequente no Brasil e em outros países da América Latina, designando pessoas que vivenciam papéis femininos, mas que não se reconhecem enquanto homens ou mulheres^1^.

No contexto mundial, travestis e transexuais representam uma parcela pequena da população geral^2^. Entretanto, a discriminação e a exclusão social impostas a essas pessoas as colocam nas piores condições de saúde e de vida^3^. Comparando com os demais grupos populacionais, as travestis e transexuais apresentam altos índices de sofrimento mental, incluindo a ideação e tentativa suicida, em virtude da discriminação e rejeição que enfrentam ao longo de suas vidas e em todos os espaços institucionais. Além disso, outros problemas de saúde são ocasionados, principalmente, pelas transformações corporais oriundas das tentativas de alinhamento do fenótipo à identidade de gênero. Assim, esses fatores acabam por gerar uma menor expectativa de vida e maiores dificuldades de acesso aos serviços de saúde por essas pessoas^3 , 4^.

No Brasil, semelhante à realidade de outros países, as travestis e mulheres transexuais são socialmente mais vulneráveis: entre lésbicas, gays, bissexuais, *queers* , intersexuais, assexuais e demais categorias de gênero e sexualidade (LGBTTQIA+), elas são as principais vítimas de violência, sobretudo de lesões corporais e de homicídios por armas de fogo^5 , 6^. Diante dessa realidade, a atenção à saúde não deve configurar-se somente em assistência aos problemas de saúde dessa população, mas também em ações amplas e concretas para acolher as travestis e as pessoas transexuais, que as auxiliem no enfrentamento à discriminação por identidade de gênero.

O sistema de saúde brasileiro, regulamentado em 1990 e denominado Sistema Único de Saúde (SUS), é majoritariamente composto por serviços públicos e gratuitos de saúde, com a complementação de alguns serviços da rede privada financiados pelo Estado^7^. Além disso, o SUS é constituído por princípios doutrinários (universalidade, equidade e integralidade), aos quais o modelo de atenção à saúde, expresso em políticas, programas, organização dos serviços e oferta de cuidados, deve voltar-se, para reconhecer os determinantes sociais do processo saúde-doença e das iniquidades em saúde^8^.

Baseando-se especialmente no princípio da equidade, as práticas de atenção à saúde devem ser realizadas de maneira mais profícua para atender as pessoas mais vulneráveis, entre essas, as travestis e as pessoas transexuais. Iniciativas direcionadas a essa população vêm sendo implementadas no SUS ao longo dos anos, como a Política Nacional de Saúde Integral LGBT, criada em 2011, e o programa Processo Transexualizador no SUS, criado em 2008 e ampliado em 2013^9^.

Na literatura científica, observa-se um aumento na publicação de artigos sobre a saúde da população LGBTTQIA+ brasileira após a criação da Política Nacional de Saúde Integral LGBT^10^, assim como de teses e dissertações sobre travestilidade, transexualidade e saúde, principalmente após a ampliação do Processo Transexualizador no SUS^11^. Ademais, é possível identificar algumas revisões integrativas sobre a saúde da população travesti e transexual no Brasil, especificamente sobre as dificuldades que essas pessoas enfrentam para ter acesso aos serviços de saúde^12 , 13^. Entretanto, não existem revisões sistemáticas que apresentem evidências sumarizadas sobre os demais aspectos relacionados à atenção à saúde da população travesti e transexual brasileira.

Considerando que o Brasil é um país de dimensão continental, com diversas e profundas desigualdades, sobretudo na efetivação do direito universal à saúde, questiona-se: como tem ocorrido a atenção à saúde para travestis e transexuais no âmbito do SUS? Em função disso, o objetivo deste estudo é sintetizar evidências científicas para caracterizar a atenção à saúde para travestis e transexuais no país.

## MÉTODOS

Trata-se de uma revisão sistemática da literatura científica, conduzida de julho de 2020 a janeiro de 2021 e atualizada em setembro de 2021. O protocolo de pesquisa^14^seguiu as recomendações do *checklist Preferred Reporting Items for Systematic Review and Meta-analysis Protocols* (Prisma-P)^15^, e está registrado na plataforma *International Prospective Register of Systematic Reviews* (Prospero), sob o código CRD42020188719.

A partir da formulação de uma pergunta-problema bem definida e de uma metodologia explícita e reprodutível, os estudos de revisão sistemática são capazes de identificar, selecionar, avaliar e sumarizar evidências científicas já disponíveis^16^. Da mesma forma, podem apontar mudanças necessárias às práticas profissionais ou recomendações para a realização de outras investigações, visando preencher lacunas do conhecimento.

O levantamento das evidências, em português e inglês, que integra esta revisão sistemática foi realizado a partir de consultas às seguintes bases de dados: *Scientific Eletronic Library Online* (SciELO), *US National Library of Medicine* (Pubmed), Literatura Latino-Americana e do Caribe em Ciências da Saúde (Lilacs) e Biblioteca Virtual em Saúde (BVS). A BVS, plataforma digital coordenada pelo Centro Latino-Americano e do Caribe de Informação em Ciências da Saúde (Bireme), reúne dados de diferentes bases eletrônicas da área da saúde, tais como: Base Regional de Informes de Avaliação de Tecnologias em Saúde das Américas (Brisa), *Littérature Scientifique em Santé* (Lissa) *, Medical Literature Analysis and Retrievel System Online* (MedLine), *Sistema de Información de la Biblioteca de la Organización Mundial de la Salud* (Wholis), *Índice Bibliográfico Español en Ciencias de la Salud* (IBECS), Base de Dados em Enfermagem (BDENF), Bibliografia Brasileira de Odontologia (BBO), entre outras.

Para conduzir a busca eletrônica dos estudos, foram utilizadas estratégias previamente definidas após diferentes tentativas, compostas por um bloco de descritores relacionados à atenção à saúde no contexto brasileiro e por um bloco de descritores relacionados às travestis e pessoas transexuais, respeitando as especificidades de cada base de dados ( Quadro 1 ).

**Quadro 1:** Estratégias de busca para as bases de dados selecionadas. Natal, Brasil, 2022.

Base de dados	Estratégia de busca
SciELO	(“atenção à saúde” OR “sistema único de saúde” OR SUS OR brasil) AND (travesti OR travestilidade OR travestismo OR transexual OR transexualidade OR transexualismo OR transgênero OR “pessoas trans”)
PubMed	(“health care” OR “health systems”) AND brazil AND (“transgender persons” OR transvestism OR transsexualism)
Lilacs	(“atenção à saúde” OR “sistema único de saúde” OR SUS OR brasil) (travesti OR travestilidade OR travestismo OR transexual OR transexualidade OR transexualismo OR transgênero OR “pessoas trans”)
BVS	(“atenção à saúde” OR “sistema único de saúde” OR SUS OR brasil) (travesti* trans*)

Nas bases SciELO, Lilacs e BVS, as estratégias de busca foram compostas por descritores extraídos do vocabulário do portal Descritores em Ciências da Saúde (DECS) e, para a busca na PubMed, foram utilizados termos equivalentes do *Medical Subject Headings* (MESH). As estratégias foram aplicadas contemplando todos os índices (título, resumo, palavras-chave e texto).

Os critérios de elegibilidade basearam-se no anagrama PECOS^16^, com as devidas adaptações ( Quadro 2 ).

**Quadro 2:** Elementos da pesquisa de acordo com o anagrama PECOS. Natal, Brasil, 2022.

Elemento	Abreviação	Descrição
Participantes	P	Travestis, mulheres e homens transexuais, profissionais da saúde, gestores da saúde.
Exposição	E	Políticas e programas de saúde, organização dos serviços de saúde, prestação do cuidado à saúde.
Comparação ou controle	C	NA
Desfecho	O	Promoção da saúde, acesso aos serviços de saúde, respostas às demandas e necessidades de saúde.
Tipos de estudo incluídos	S	Estudos originais com abordagem qualitativa ou quantitativa.

NA: Não se aplica.

Fonte: Autoria própria. Adaptado de Galvão e Pereira ^16^ .

A adaptação no anagrama PECOS concerniu ao elemento comparação ou controle, não sendo considerado para os critérios de inclusão deste estudo. Portanto, foram incluídos na revisão os artigos resultantes de estudos originais, com abordagem qualitativa ou quantitativa, sobre os aspectos relacionados à atenção à saúde para a população travesti e transexual no Brasil, e nos quais os sujeitos fossem travestis, pessoas transexuais, profissionais da saúde ou gestores da saúde. Ademais, os estudos incluídos foram aqueles publicados de forma completa em periódicos científicos a partir de 1990, ano de regulamentação do SUS, independentemente do idioma.

Foram excluídos os estudos que abordavam a atenção à saúde para travestis e transexuais somente no sistema de saúde privado ou conjuntamente com os aspectos relacionados à atenção à saúde para as demais pessoas do grupo LGBTTQIA+, e os estudos classificados como resumos publicados em anais, editoriais, artigos reflexivos, análises documentais, revisões de literatura, manuais técnicos, capítulos, livros, monografias, dissertações e teses.

As publicações foram recuperadas por um único pesquisador e, logo depois, codificadas e organizadas numa planilha eletrônica para posterior remoção das duplicatas. Em seguida, considerando os critérios de inclusão e exclusão adotados, foi realizada triagem a partir da leitura dos títulos e resumos dos arquivos, para selecionar aqueles que poderiam ser incluídos na revisão sistemática e que seriam lidos na íntegra.

A etapa de leitura integral foi realizada com a finalidade de eleger aqueles estudos com forte potencial de inclusão na revisão sistemática. Por meio da busca manual e de forma complementar, as listas de referências dos artigos triados também foram consultadas para identificar as possíveis perdas durante a busca eletrônica.

Os artigos selecionados foram lidos novamente na íntegra para que a qualidade metodológica deles fosse avaliada por dois pesquisadores previamente calibrados, visando garantir uniformidade na avaliação crítica. Isso aconteceu por meio das ferramentas de avaliação crítica do *Joanna Briggs Institute* (JBI), sendo utilizado um *checklist* específico para estudos com abordagem qualitativa^17^, uma vez que todos os artigos elegíveis possuíam essa abordagem metodológica.

Com base no *checklist* apresentado por Lockwood *et al* .^17^, assim como na classificação proposta por Almeida *et al.* ^18^, o risco de viés dos artigos foi classificado entre alto (com até 49% de respostas afirmativas), moderado (respostas afirmativas entre 50% e 69%) e baixo (70% ou mais de respostas afirmativas). Neste estudo de revisão, a síntese final foi composta somente por artigos com baixo risco de viés.

Todo o processo de triagem, elegibilidade e avaliação da qualidade metodológica dos artigos foi realizado por dois pesquisadores independentes. Em caso de dúvida ou discordância, os pesquisadores se reuniram virtualmente para discutir e estabelecer um consenso.

A síntese dos achados foi realizada por meio da narrativa formal e com o auxílio da construção de tabelas contendo alguns dados dos artigos: título, autoria, ano de publicação, objetivo, amostra/público-alvo, local de realização do estudo (Unidade Federativa do Brasil), tipo de estudo, abordagem metodológica, resultados e conclusões.

Os resultados dos estudos incluídos na revisão sistemática foram agrupados categoricamente, de acordo com as suas aproximações temáticas, e interpretados com o suporte da literatura científica que trata sobre a questão aqui investigada. Por fim, a redação final do presente manuscrito obedeceu às recomendações do *checklist Prefered Reporting Items for Systematic Reviews and Meta-analyses* (Prisma)^19^.

## RESULTADOS

Por meio da busca nas bases de dados de interesse desta revisão sistemática, foi possível recuperar 1.164 títulos. Após a remoção das duplicatas, da triagem a partir da leitura dos títulos e resumos, da inclusão de outras publicações mediante a verificação das referências dos artigos triados, e da atualização de busca, 45 artigos foram submetidos à leitura integral. Desses, 24 foram submetidos à avaliação da qualidade metodológica e 15 foram incluídos na síntese dos resultados, sendo 14 artigos publicados em português e um artigo publicado em inglês ( Figura 1 ).

**Figura 1:** Fluxograma do processo de identificação, triagem, elegibilidade e inclusão dos artigos da revisão sistemática. Natal, Brasil, 2022.

Fonte: Autoria própria. Adaptado de Flor et al. ^20^ .

Os 15 artigos incluídos nesta revisão sistemática não atendem o sexto critério do *checklist* utilizado para nortear a avaliação da qualidade metodológica^17^, ou seja, não apresentam informações explícitas acerca da localização cultural ou teórica dos(as) pesquisadores(as) e sua possível influência no estudo ( Quadro 3 ).

**Quadro 3:** Avaliação da qualidade metodológica dos artigos incluídos na revisão sistemática, conforme critérios do *checklist* utilizado ^17^ e da classificação proposta por Almeida et al. ^18^ . Natal, Brasil, 2022.

Artigos	Critérios de avaliação do checklist										Risco de viés (%)^b^

	1	2	3	4	5	6	7	8	9^a^	10	
Oliveira e Romanini ^21^	A	A	A	A	A	B	A	A	A	A	Baixo (90%)
Rigolon et al. ^22^	A	A	A	A	A	B	A	A	A	A	Baixo (90%)
Sousa e Iriart ^23^	A	A	A	A	A	B	C	A	A	A	Baixo (80%)
Moraes e Silva ^24^	A	A	A	A	A	B	B	A	A	A	Baixo (80%)
Sehnem et al. ^25^	B	A	A	A	A	B	B	A	A	A	Baixo (70%)
Amorim e Teixeira ^26^	C	A	A	A	A	B	B	A	A	A	Baixo (70%)
Sehnem et al. ^27^	B	A	A	A	A	B	B	A	A	A	Baixo (70%)
Almeida et al. ^28^	B	A	A	A	A	B	B	A	A	A	Baixo (70%)
Souza et al. ^29^	C	A	A	A	A	B	B	A	A	A	Baixo (70%)
Oliveira et al. ^30^	B	A	A	A	A	B	B	A	A	A	Baixo (70%)
Sevelius et al. ^31^	A	A	A	A	A	B	C	A	C	A	Baixo (70%)
Lovison et al. ^32^	B	A	A	A	A	B	B	A	A	A	Baixo (70%)
Ferreira et al. ^33^	C	A	A	A	A	B	B	A	A	A	Baixo (70%)
Hanauer e Hemmi ^34^	A	A	A	A	A	B	C	A	C	A	Baixo (70%)
Silva et al. ^35^	B	A	A	A	A	B	B	A	A	A	Baixo (70%)

Legenda: A = sim, B = não, C = não está claro.

^a^Para atendimento do critério, considerou-se a inclusão do número do parecer de aprovação ética no texto do artigo.

^b^Porcentagem de respostas afirmativas.

Fonte: Autoria própria. Adaptado de Flor et al. ^20^

Em estudos com abordagens qualitativas, as crenças e valores dos(as) pesquisadores(as), assim como suas orientações teóricas, podem influenciar a condução da pesquisa, desde a coleta dos dados até a divulgação dos resultados. Portanto, é importante que os artigos que prezem pelo rigor metodológico explicitem as possíveis influências dos(as) pesquisadores(as) no estudo, ou as estratégias adotadas para diminuir essas potenciais influências^17^.

A síntese dos estudos incluídos ( Quadro 4 ) revela que 10 artigos, representando a maioria, foram publicados entre 2017 e 2019. As pesquisas que originaram tais artigos foram desenvolvidas no Sul e Sudeste do Brasil, cinco deles em cada região. Logo, não foram incluídos os artigos decorrentes de estudos realizados na região Norte.

**Quadro 4:** Síntese das principais características dos artigos incluídos na revisão sistemática. Natal, Brasil, 2022.

Autoria	Ano	UF	Sujeitos da pesquisa	Objetivo	Resultados	Conclusões
Oliveira e Romanini ^21^	2020	RS	6 mulheres transexuais.	Compreender a forma como se constroem as trajetórias das pessoas transgênero nas políticas públicas de saúde em um município do interior do Rio Grande do Sul.	As mulheres trans possuem maior aproximação com o ambulatório transexualizador. Contudo, se constrói uma relação frágil nesse ambiente, pois as usuárias se sentem constantemente avaliadas quanto à “veracidade” de sua transexualidade, tornando-se passivas no processo de produção de saúde em função da superioridade do saber médico e reprodução de protocolos invisíveis.	O percurso das mulheres trans nas políticas públicas de saúde é pautado pela invisibilidade, acarretando a construção de linhas de cuidado que não condizem com as reais necessidades desse grupo populacional. É preciso incentivar a integração ensino-serviço, visando construir uma interlocução entre o conhecimento científico sobre transexualidade e as vivências das pessoas trans, para qualificar os trabalhadores da saúde e, consecutivamente, a atenção à saúde das mulheres trans.
Rigolon et al. ^22^	2020	SP	1 travesti, 1 mulher transexual e 2 homens transexuais.	Compreender as histórias de vida e o itinerário de travestis e transexuais nos serviços de saúde.	Os resultados foram apresentados em dois principais temas: “gênero e sexualidade nas histórias de vida” e “as trajetórias nos serviços de saúde”. Os relatos revelam os desafios enfrentados por pessoas trans no processo de reconhecimento da identidade de gênero e os dilemas para acessarem os serviços de saúde, afastando-as dos espaços de cuidado.	O método da História Oral pode ampliar o conhecimento acerca das necessidades e demandas de saúde de travestis e transexuais. Além disso, os resultados da pesquisa podem auxiliar profissionais e gestores da saúde na tomada de decisões e no cuidado integral a essas pessoas.
Sousa e Iriart ^23^	2018	BA	10 homens transexuais.	Compreender as necessidades e as demandas de saúde dos homens trans, considerando tratar-se de um passo primeiro para viabilizar caminhos pelos quais estratégias de cuidado possam ser pensadas.	A transfobia, um mecanismo estrutural e institucional de violência e aversão às pessoas trans, determina as práticas de cuidado e o desenvolvimento de estratégias para atender às necessidades e demandas de saúde dos homens trans. Estas, por sua vez, são apresentadas em três categorias: “despatologização das vivências trans”, “modificações corporais” e “atendimentos ambulatoriais”.	A transfobia influencia de maneira negativa o modo de viver dos homens trans, agudizando as suas necessidades e demandas de saúde. É necessário desenvolver estratégias intersetoriais para combater a transfobia e garantir o direito à saúde e à vida dos homens trans.
Moraes e Silva ^24^	2020	RN	7 travestis.	Identificar concepções, desejos, receios e sugestões quanto ao cuidado humanizado em saúde dirigido às pessoas travestis no âmbito da atenção primária.	As travestis enfrentam dificuldades para acessar e usar os serviços de saúde. Para superá-las e mudar o cuidado em saúde das travestis, são apontadas algumas sugestões: capacitação dos profissionais de saúde, diálogo com o movimento social, campanhas de divulgação e aproximação com o significado da vivência de ser travesti.	Para que a atenção primária à saúde seja um espaço acolhedor e humanizado para as travestis, é preciso primar pelo respeito e pela garantia de direitos. É preciso resgatar a promoção do cuidado, algo fundamental nas práticas de trabalho em saúde, respeitando as diferenças e exercitando o altruísmo. É urgente um trabalho efetivo de mudanças na formação dos profissionais de saúde.
Sehnem et al. ^25^	2017	RS	10 enfermeiros.	Conhecer a assistência em saúde às travestis, sob a ótica dos enfermeiros, na atenção primária.	Surgiram duas categorias temáticas: “as fragilidades no atendimento às demandas das travestis na atenção primária à saúde” e “o acolhimento como prática integradora na atenção primária em saúde”. Os enfermeiros não se sentem preparados para acolherem as travestis e acreditam que elas não buscam as Unidades Básicas de Saúde com receio de serem constrangidas.	As poucas ações de saúde direcionadas às travestis ocorreram de maneira isolada e fragmentada. Para superação dos desafios, recomenda-se a implementação da política de saúde LGBT no SUS, a educação permanente dos trabalhadores da saúde e o fortalecimento do acolhimento para potencializar o vínculo entre profissionais e usuárias travestis.
Amorim e Teixeira ^26^	2017	RJ	5 travestis.	Discutir o atendimento das necessidades em saúde das travestis nos serviços de saúde da atenção primária.	Para as entrevistadas, a condição de saúde está atrelada às ações de promoção da saúde. Na busca por modificações corporais, as travestis fazem uso de hormônios femininos sem acompanhamento, acarretando o surgimento de outras demandas, como complicações cardiovasculares. Ao buscarem as Unidades Básicas de Saúde (UBS), as travestis enfrentam desrespeito ao nome social e outras violações, o que as distanciam desses serviços de saúde, aproximando-as de práticas alternativas ou clandestinas para o autocuidado.	A atenção primária ainda é frágil para atender as necessidades e demandas de saúde das travestis. Recomenda-se implementar uma política pública de saúde que considere as especificidades de saúde das travestis para a promoção da atenção integral à saúde; incluir conteúdos sobre o cuidado às travestis nos cursos da área da saúde e desenvolver ações educativas permanentes, sobre acolhimento e humanização, aos profissionais de saúde inseridos na atenção primária.
Sehnem et al. ^27^	2017	RS	10 enfermeiras(os).	Conhecer as percepções das(os) enfermeiras(os) acerca das travestis e o preparo técnico-científico para assisti-las.	Os dados foram apresentados em duas categorias temáticas: “travesti: significados construídos sob a ótica de enfermeiras(os)” e “(des)preparo técnico-científico: repercussões no cuidado prestado às travestis”. As(os) enfermeiras(os) desconhecem o significado de ser travesti e as políticas públicas voltadas à população LGBT, sendo um dos motivos apontados para esse desconhecimento a frágil abordagem das questões de gênero e sexualidade durante a formação acadêmica.	Para o cuidado às travestis, é necessário repensar as políticas públicas de saúde para essa parcela da população, garantir o acesso e o acolhimento nos serviços de saúde, além de qualificar o preparo técnico-científico dos profissionais de saúde acerca das questões de gênero e sexualidade.
Almeida et al. ^28^	2018	RJ	13 enfermeiros.	Identificar a formação dos enfermeiros residentes para o cuidar qualificado de pessoas transexuais e analisar o processo de cuidar de enfermagem dessa clientela, na perspectiva do residente de enfermagem.	Os depoimentos foram apresentados em duas categorias: “a formação do enfermeiro e o cuidar de pessoas transexuais” e “o processo do cuidar de enfermagem na perspectiva do residente”. Na visão dos residentes, a formação do profissional de enfermagem não está atenta para as particularidades das diferentes identidades de gênero, incluindo as das pessoas transexuais. Assim, o despreparo desses profissionais para prestar um atendimento qualificado, frente às necessidades das pessoas transexuais, será perpetuado.	Evidenciou-se o despreparo dos profissionais de enfermagem para o atendimento das demandas sociais e de saúde da população transexual, justificado, sobretudo, pela ausência de conteúdos curriculares sobre transexualidade durante a formação profissional.
Souza et al. ^29^	2014	RS	49 travestis.	Apresentar os itinerários terapêuticos de travestis residentes em Santa Maria, município da região central do Rio Grande do Sul.	Constatou-se que as travestis buscam por caminhos múltiplos para obterem cuidados com a saúde, inclusive fora dos serviços institucionalizados de saúde. Para uma melhor compreensão dos itinerários terapêuticos das travestis, os resultados do estudo foram apresentados em quatro categorias: “a trajetória da construção travesti”, “itinerário de cuidados nos espaços percorridos pelas travestis”, “o itinerário nos serviços públicos de saúde” e “o itinerário na religião afro, o batuque”.	O estudo revelou que as travestis evitam os serviços públicos de saúde, pois quando buscam sempre são vítimas de discriminação. Tais serviços de saúde não estão preparados para atenderem as suas necessidades e demandas. Portanto, os itinerários terapêuticos das travestis de Santa Maria, Rio Grande do Sul, são constituídos por outros cenários e práticas de saúde, para além dos espaços institucionais do SUS, tais como espaços de moradia, pontos de prostituição, nos espaços públicos e nas “casas de santo”.
Oliveira et al. ^30^	2019	DF	2 pessoas transexuais.	Compreender como os movimentos sociais trans do Distrito Federal entendem o direito à saúde.	Compreende-se que o direito à saúde está relacionado à três unidades temáticas: “direito de existir”, como a busca por direitos humanos básicos (uso e respeito do nome social); “direito à equidade”, uma vez que os serviços de saúde devem estar preparados para atenderem as necessidades específicas da população trans; e “implementação do ambulatório trans e a participação social”, evidenciando a importância do controle social para a efetivação do direito à saúde.	A luta pelo reconhecimento das necessidades específicas da população trans e a conquista de equipamentos sociais para atender de forma satisfatória essa população, como o ambulatório trans, são iniciativas que podem proporcionar um diálogo permanente entre as pessoas trans e os profissionais de saúde para a produção do cuidado específico e qualificado, reverberando na efetivação do direito à saúde e à cidadania.
Sevelius et al. ^31^	2019	RJ	36 mulheres transexuais.	Explorar como o contexto social de estigma e transfobia afetam o acesso das mulheres trans brasileiras e suas preferências por método de prevenção de HIV e programação de cuidados guiados pelo Modelo de Afirmação de Gênero.	Os resultados retratam a discriminação nos serviços de saúde, desde o desrespeito do nome social até a concepção prévia de que essas usuárias são pessoas que vivem com HIV. As mulheres trans possuem incertezas sobre a eficácia e o fornecimento da Profilaxia Pré-Exposição (PrEP), e não estão emocionalmente preparadas para encarar um possível resultado positivo para HIV. As atividades em grupo podem explorar outras questões, para além do HIV, garantindo a possibilidade de convivência entre pessoas soropositivas ou não. As entrevistadas demonstram um sentimento de confiança ao se depararem com serviços de saúde que possuem trabalhadores(as) transexuais entre a equipe.	Os dados apoiam fortemente uma abordagem de afirmação de gênero para a saúde trans, na qual o programa de HIV para mulheres transexuais não é limitado às estratégias individuais e biomédicas, como maior ingestão de PrEP ou aumento do índice de testes. É preciso afirmar as subjetividades diversas das mulheres transexuais e dar suporte à sua autonomia, à medida que cultivam a saúde e o empoderamento, tanto para elas mesmas quanto para suas comunidades.
Lovison et al. ^32^	2019	SC	4 mulheres transexuais e 1 travesti.	Conhecer a percepção de travestis e transexuais residentes em Chapecó, Santa Catarina, acerca do acesso e assistência em saúde.	A busca pelo atendimento nos serviços públicos de saúde não é prioridade para as entrevistadas. Elas acabam por optar pela automedicação ou arcando com os custos da assistência privada. Isso decorre dos atos discriminatórios que elas enfrentam nos referidos serviços, principalmente o desrespeito ao nome social e o desconhecimento dos profissionais de saúde sobre as políticas e programas de saúde para essa população específica.	O acesso e assistência à saúde são limitados e fragmentados em virtude do número reduzido de profissionais e serviços de saúde que atendam as demandas gerais da população do município de Chapecó, e do despreparo do sistema municipal de saúde para atender as demandas específicas da população trans. As mulheres trans não possuem os direitos básicos atendidos, como o direito à saúde; não recebem informações sobre processos e procedimentos em saúde e são discriminadas por profissionais de saúde e demais usuários dentro dos serviços de saúde.
Ferreira et al. ^33^	2017	PI	6 travestis.	Analisar e compreender as vivências de travestis acerca da atenção à saúde no SUS em Teresina, Piauí.	Surgiram duas categorias temáticas: “fragilidades no atendimento às travestis” e “especialização do cuidado às travestis”. A primeira categoria revela situações de preconceito nos serviços de saúde em função das mudanças corporais, gerando insegurança por parte dessa população ao recorrer aos serviços. A segunda categoria sugere que a criação de serviços específicos para travestis poderia minimizar situações de vexame.	Evidenciou-se que há a necessidade de maior integração entre os diversos segmentos sociais e os serviços de saúde, a qualificação dos profissionais para garantir o acolhimento às travestis e a reflexão urgente sobre o caráter discriminatório dos serviços especializados implantados pelo SUS, mesmo sendo uma porta para as travestis adentrarem o sistema de saúde.
Hanauer e Hemmi ^34^	2019	MG	4 homens transexuais e 3 mulheres transexuais.	Descrever os caminhos percorridos por transexuais, visando conhecer seus itinerários na busca por atendimento às suas necessidades e demandas em saúde.	As diferentes trajetórias apresentadas estão relacionadas ao processo de identificação como pessoa transexual e à influência das redes sociais, assim como aos serviços de saúde nesse percurso. As redes de cuidados em saúde foram reconhecidas como central na vida das pessoas trans entrevistadas, sobretudo no que diz respeito ao acesso às modificações corporais.	A busca pela identidade de gênero desejada é atravessada por inúmeras dificuldades, como a não aceitação familiar e social e a negação do direito à saúde quando buscam os serviços do SUS. A transexualidade parece ser incompreendida por parte das pessoas do convívio social e por profissionais e gestores das redes de atenção à saúde.
Silva et al. ^35^	2014	RN	12 enfermeiras (os).	Analisar a prática e o conhecimento de enfermeiros(as) da Estratégia Saúde da Família (ESF) no tocante à assistência à população travesti.	Os resultados do estudo são apresentados a partir de três eixos discursivos. O primeiro eixo diz respeito à construção da identidade travesti, como os(as) profissionais entrevistados(as) compreendem essa identidade de gênero e como desenvolvem práticas de enfermagem para esse grupo populacional. No segundo eixo, os(as) enfermeiros(as) revelam não terem atendido travestis na atenção primária ou não saberem identificá-las. O terceiro eixo apresenta iniciativas de alguns profissionais para melhor acolher e atender as travestis, como o respeito ao nome social.	Os(as) enfermeiros(as) demonstraram que a prática e o conhecimento na assistência às travestis são limitados, não conseguindo identificá-las pelo gênero de escolha, julgando-as apenas por suas características físicas e aparentes. Em virtude dessa invisibilidade, realizam apenas um atendimento curativista, sem considerar as subjetividades das travestis e o respeito ao nome social, desqualificando o cuidado a esse público específico.

UF: Unidade Federativa do Brasil.

Em sua totalidade, os artigos possuem a abordagem qualitativa como única característica metodológica. Além disso, seis dos artigos tiveram travestis e mulheres transexuais como sujeitos das pesquisas e três foram desenvolvidos com profissionais de saúde, especificamente com enfermeiras(os), focando na percepção destes sobre a atenção à saúde para travestis e transexuais.

Ainda de acordo com a síntese contida no Quadro 4 , oito artigos (a maioria) possuem como foco de análise o acesso de travestis e transexuais aos serviços de saúde e o cuidado ofertado a esse público. A partir das aproximações temáticas identificadas nos resultados dos artigos, os achados desta revisão foram agrupados em seis categorias, apresentadas a seguir.

### Possibilidades para Transformar a Atenção à Saúde

No que tange à atenção à saúde para travestis e transexuais no Brasil, os artigos revelam, de forma quase unânime, as dificuldades existentes para a efetivação dos princípios basilares do SUS. Diante do diagnóstico acerca dessas dificuldades, que serão abordadas nas categorias seguintes, são apontadas possibilidades para transformar esse modelo de atenção à saúde, tais como: desenvolvimento de estratégias intersetoriais para o combate à discriminação contra travestis e transexuais, inclusive nos serviços de saúde^23 , 33^; diálogo constante entre a gestão do SUS e os segmentos sociais que representam travestis e pessoas transexuais, na perspectiva de compreender e respeitar as especificidades de saúde dessa parcela da população^24 - 26 , 30 , 33^; efetivação da Política Nacional de Saúde Integral LGBT, garantindo o acesso de travestis e transexuais aos diferentes estabelecimentos do SUS^25 - 27^; estratégias de divulgação do significado de ser travesti ou transexual para a comunidade em geral, visando afirmar as subjetividades e respeitar as necessidades dessas pessoas^24 , 31^; revisão das práticas de trabalho em saúde, envolvendo o resgate da promoção do cuidado, altruísmo pelos profissionais de saúde, desenvolvimento da autonomia de travestis e transexuais e inclusão de trabalhadores(as) transexuais nas equipes de saúde^24 , 25 , 31^; e mudanças na formação profissional em saúde, incentivando a integração ensino-serviço-comunidade, a inclusão de conteúdos sobre gênero e sexualidade nos currículos dos cursos da área da saúde e o desenvolvimento de ações de educação permanente acerca do cuidado humanizado às travestis e transexuais para os trabalhadores do SUS^21 , 24^.

### Travestifobia e Transfobia: Violações Dentro e Fora do SUS

No decorrer de suas vidas, travestis bem como mulheres e homens transexuais são vítimas de atitudes preconceituosas, reflexo de uma violência estrutural (travestifobia e transfobia) causada pelo simples fato de se tratar de um grupo que rompe com o padrão hegemônico, que tenta definir o gênero somente pela anatomia dos órgãos genitais.

Essa aversão surge logo no seio familiar, quando travestis e transexuais em busca do reconhecimento de suas identidades de gênero passam a não serem aceitos pela própria família. Essa não aceitação ocorre igualmente por parte de pessoas do convívio social^22 , 34^. Além disso, mais do que estrutural, a travestifobia e a transfobia são violências também institucionais, ou seja, estão presentes em instituições como o SUS.

Portanto, o reconhecimento das violências sofridas por travestis e transexuais deve fazer parte do processo de trabalho na área da saúde, a fim de tentar acolher e dar suporte às vítimas. Porém, atos discriminatórios, como o desrespeito ao nome social e a incompreensão sobre travestilidade e transexualidade por parte dos profissionais e gestores da saúde^31 , 34^, influenciam negativamente as práticas de atenção à saúde para esse público específico.

Com isso, travestis e transexuais sentem insegurança ao recorrerem aos serviços de saúde^33 , 34^, chegando a evitá-los^22 , 29^. No caso de homens transexuais, como relata Sousa e Iriart^23^a partir de um estudo realizado em Salvador, na Bahia, a transfobia institucional impede o acesso deles aos serviços de saúde que atendam às suas necessidades e demandas específicas, incluindo o acompanhamento hormonal para as modificações corporais.

Dados de um estudo com mulheres transexuais brasileiras revelam que a concepção prévia dos profissionais de saúde de que elas conviveriam com o Vírus da Imunodeficiência Humana (HIV), entre outras Infecções Sexualmente Transmissíveis (IST), faz com que essas usuárias deixem de buscar pelos serviços do SUS, considerados discriminatórios por essas mulheres^31^. Além disso, o preconceito e estigma dos profissionais de saúde relacionados a elas acaba por afastá-las do acesso às estratégias de prevenção combinada do HIV, como o uso da PrEP^31^.

### Despreparo Profissional para a Atenção às Travestis e Transexuais

Os achados revelam que profissionais de saúde, em especial profissionais de enfermagem, não estão devidamente qualificados para acolher e cuidar de travestis e pessoas transexuais nos serviços de saúde^25 , 27 , 28 , 35^. Na visão deles, o despreparo para a atenção à saúde desse público específico é decorrente da frágil abordagem das questões sobre diversidade de gênero e sexualidade durante o processo de formação profissional^27^.

Em decorrência disso, alguns enfermeiros(as) desconhecem as subjetividades de uma pessoa travesti ou transexual e suas necessidades e demandas de saúde^27 , 28^. Ademais, a ausência de ações educativas permanentes, que divulguem as diretrizes da Política Nacional de Saúde Integral LGBT e que qualifiquem os cuidados às travestis e transexuais no SUS, faz com que profissionais de enfermagem perpetuem estigmas e continuem a limitar o cuidado a essas pessoas, desrespeitando-as e pautando as suas práticas de atenção somente em ações curativas^35^.

### Busca por Alternativas para a Atenção à Saúde

Devido ao despreparo profissional, e aos atos discriminatórios vivenciados no interior dos estabelecimentos do SUS já apontados nos resultados deste artigo, travestis e transexuais evitam buscar por cuidados nesses espaços institucionais^22 , 29 , 32 - 34^. Diante dessas violações, uma minoria de travestis e transexuais optam pela assistência privada, porém, a maioria não possui condições financeiras para arcar com as despesas de um plano de saúde, tornando-se dependentes de práticas alternativas de autocuidado, do apoio de pessoas da rede social ou espaços religiosos de matriz africana, ou de transformações corporais sem o devido acompanhamento profissional^26 , 29 , 32^.

Nesse contexto, as transformações corporais, como a hormonioterapia inapropriada, o enxerto de silicone industrial e a automutilação mamária, podem ocasionar outros problemas de saúde, como complicações cardiovasculares e estéticas. Isso caracteriza claramente mais um fator de risco à vida de travestis e pessoas transexuais^26^.

### Direito à Saúde para Travestis e Transexuais: Utopia ou Realidade?

Segundo a Constituição Federal do Brasil, o acesso à saúde é um direito básico de todas as pessoas, garantido mediante políticas públicas que visam diminuir o risco de doenças e agravos, assim como permitir o acesso universal e igualitário às ações e serviços de saúde^36^. Entretanto, para minorias sociais brasileiras, como travestis e transexuais, esse direito fundamental não tem sido real.

O direito à saúde para travestis e transexuais, ou a não concretude dele, pode ser compreendido por três aspectos: o direito de existir, considerando que essas pessoas ainda lutam para terem seus nomes sociais respeitados, inclusive no SUS; direito à equidade, pois os serviços de saúde ainda não estão preparados para atenderem travestis e transexuais; e a participação social, porque travestis e transexuais precisam exercer o controle social para acessar e permanecer nos serviços de saúde, e para reivindicar a criação de serviços específicos para a oferta de procedimentos transexualizadores^30 , 32 , 34^.

### O Processo Transexualizador: Avanços e Desafios

No Brasil, os procedimentos transgenitalizadores, a título experimental, estão autorizados pelo Conselho Federal de Medicina (CFM) desde 1997, sendo incorporados ao SUS em 2008, quando o Ministério da Saúde fundou o programa Processo Transexualizador e reconheceu formalmente que as transformações corporais também são necessidades de saúde da população travesti e transexual^9^. Tais procedimentos, assim como a criação dos serviços habilitados para ofertá-los, representam um avanço na promoção da saúde de travestis e transexuais.

Por isso, espera-se que os serviços habilitados para a oferta dos procedimentos previstos no Processo Transexualizador sejam espaços acolhedores e ausentes de discriminação, pois são constituídos por equipes multiprofissionais e devidamente qualificadas para cuidar de travestis e transexuais de forma humanizada^33^. Todavia, nota-se um vínculo frágil entre profissionais de saúde e usuários(as), em especial com mulheres trans^21^.

Segundo Oliveira e Romanini^21^, tal situação pode ser justificada pela existência de uma superioridade médica e de um protocolo assistencial, com duração de dois anos, que trata a transição da identidade de gênero como transtorno, fazendo desse diagnóstico uma condição para se ter acesso aos serviços de saúde específicos dessa parcela da população. Com isso, nesses serviços, as travestis e transexuais são constantemente indagadas(os) quanto à veracidade das suas identidades de gênero e de suas intenções, não permitindo a elas/eles uma autonomia na produção de cuidados.

## DISCUSSÃO

O modelo de atenção à saúde consiste em um conjunto de saberes e combinações de recursos (financeiros, tecnológicos, humanos etc.) para atender a necessidades de saúde individuais e coletivas^37^. Portanto, analisar o modelo de atenção à saúde, seja para a população geral ou para grupos específicos, como o grupo de travestis e pessoas transexuais, implica em compreender quais são as políticas públicas de saúde existentes para mitigar ou anular as iniquidades e como essas políticas refletem em programas, ações, serviços e pesquisas para tal^38^.

Diante dos resultados da presente revisão sistemática, percebe-se que a atenção à saúde para travestis e transexuais no território brasileiro ainda é excludente, fragmentada, centralizada no cuidado especializado e pautada por ações curativas, assemelhando-se aos modelos de atenção que antecederam o SUS e que são fortemente criticados desde a década de 1970, a partir do movimento da Reforma Sanitária Brasileira. Na ocasião, as críticas evidenciavam a importância de profundas transformações não somente no sistema de saúde, para que a atenção fosse humanizada, equânime e integral, garantindo o acesso universal a todos e todas^38^.

Segundo Arán *et al* .^39^, apesar da transexualidade ser um fenômeno reconhecido desde o final do século XIX, as questões relacionadas às identidades transgêneras na saúde pública brasileira passaram a ter visibilidade somente a partir de 1979, com a possibilidade de intervenções médicas, quando se consultou, pela primeira vez, o CFM sobre a mamoplastia de aumento em pessoas transexuais.

Avançando no tempo, e reconhecendo a existência de inúmeros debates que ocorreram acerca do assunto, inclusive no campo jurídico, em 1997 – como já foi abordado neste artigo –, os procedimentos transgenitalizadores foram autorizados pelo CFM e incorporados ao SUS 11 anos depois^9^. Cabe ressaltar que a existência desses procedimentos no SUS é uma iniciativa fundamental não só para atender uma necessidade de travestis e transexuais, mas, também, para auxiliar na construção de subjetividades e identidades negligenciadas pela sociedade e pelo Estado.

Entretanto, críticas são feitas aos protocolos assistenciais estabelecidos pelo Ministério da Saúde, por meio de normativas^40 , 41^, para que travestis e transexuais possam usufruir desses serviços de saúde especializados, principalmente o acompanhamento psiquiátrico por no mínimo dois anos a fim de se confirmar o diagnóstico de transexualidade. Ao mesmo tempo em que esse diagnóstico representa a conquista do direito à saúde de travestis e transexuais, ele contribui para a permanência do estigma sobre essas pessoas, pois não considera as questões pessoais e históricas de cada sujeito, atribuindo o reconhecimento da identidade de gênero a um procedimento psiquiátrico e normalizador^39^.

Ademais, o acesso aos procedimentos transgenitalizadores não é a única necessidade de saúde de travestis e transexuais no Brasil. Para além do acesso a esses procedimentos, é necessário promover o combate à discriminação por identidade de gênero para que travestis e transexuais possam acessar dignamente qualquer espaço, como os espaços de promoção da saúde, sem serem vítimas de violências^26^.

Se o acesso aos procedimentos transgenitalizadores fosse a única necessidade de saúde de travestis e transexuais brasileiras, ele seria muito desigual e longe de ser atendido, pois no país só existem dez serviços habilitados pelo Ministério da Saúde para a oferta dos procedimentos previstos no programa Processo Transexualizador, sendo a maioria localizada no Sudeste e nenhum na região Norte^9^.

Os achados desta revisão sistemática também revelam possíveis caminhos para transformar o atual modelo de atenção à saúde para travestis e transexuais no SUS: seriam interessantes o desenvolvimento de estratégias intersetoriais para o combate à discriminação contra travestis e transexuais^23 , 33^, o diálogo constante entre a gestão do SUS e os segmentos sociais que representam travestis e pessoas transexuais^24 - 26 , 30 , 33^, a efetivação da Política Nacional de Saúde Integral LGBT^25^, estratégias de divulgação do significado de ser travesti ou transexual para a comunidade em geral^24 , 31^, a revisão das práticas de trabalho em saúde^24 , 25 , 31^e as mudanças na formação profissional em saúde^21 , 24^.

Considerando essas possibilidades, destaca-se aqui a relevância da participação dos movimentos sociais representativos desse segmento na formulação e condução das políticas públicas de saúde, por meio do exercício da cidadania e do controle social. No processo de construção do SUS e das suas políticas de saúde, aglutinando-se com a luta social pela redemocratização do país, o movimento LGBTTQIA+ teve uma importante atuação, colocando em pauta a diversidade sexual e de gênero como marcadores sociais e determinantes estruturais do processo saúde-doença^42 , 43^.

Como fruto da atuação dos movimentos sociais LGBTTQIA+ na saúde, destacam-se a criação do Programa Brasil sem Homofobia e do Comitê Técnico de Saúde da População LGBT no Ministério da Saúde em 2004, a realização do I Seminário Nacional de Saúde da População LGBT em 2007, a instituição do Processo Transexualizador no SUS em 2008, a normatização do uso do nome social de travestis e transexuais no SUS em 2009, a formulação da Política Nacional de Saúde Integral LGBT etc.^44^

Concluindo, a atenção à saúde para travestis e transexuais no Brasil não corresponde ao modelo de atenção à saúde preconizado no arcabouço jurídico-legal do SUS. Portanto, é necessário desenvolver estratégias para lidar com questões específicas de travestis e transexuais, com destaque para as vulnerabilidades que permeiam as vidas dessas pessoas.

Para além da garantia da assistência à saúde, é preciso proporcionar oportunidades de acesso à educação, emprego, moradia, alimentação etc. Essas ações não devem partir somente de travestis e transexuais, mas daqueles(as) que acreditam na potencialidade do SUS e em uma sociedade igualitária e democrática.

No que tange à atuação de profissionais de saúde frente às travestis e transexuais, essa análise deteve-se aos profissionais de enfermagem, pois os estudos recuperados e incluídos nesta revisão abordavam somente essa categoria profissional. Para não induzir que o despreparo profissional para a atenção à saúde de pessoas travestis e transexuais no sistema de saúde brasileiro é uma especificidade dos(as) profissionais de enfermagem, e considerando que o trabalho no SUS deve ser interprofissional e colaborativo, recomenda-se mais estudos que investiguem a forma na qual outros profissionais da saúde lidam com travestis e pessoas transexuais.

Ao mesmo tempo em que são tecidas críticas, é importante destacar que essa análise acerca do trabalho de enfermeiras(os), em prol da atenção às travestis e transexuais, pode ser resultante de avanços nas pesquisas sobre diversidade de gênero na área da enfermagem. Todavia, conforme demonstrado nos resultados aqui apresentados, ainda é necessário mudar práticas de trabalho, permitindo uma transformação significativa no cotidiano dos espaços de atenção à saúde para travestis e transexuais.

Como limitações do estudo, aponta-se a possibilidade de alguns artigos não utilizarem os descritores adotados nas estratégias de busca da presente revisão e da existência de outros artigos indexados em bases de dados não consultadas. Todavia, para contornar essa possível situação, buscas manuais foram realizadas.

Além disso, outras limitações referem-se à não inclusão de estudos desenvolvidos na região Norte do Brasil, considerando que o SUS está presente em todo o território nacional, e à escolha dos autores em excluir da revisão sistemática artigos que analisavam a atenção à saúde para travestis e transexuais conjuntamente com os aspectos relacionados à atenção à saúde para as demais pessoas do grupo LGBTTQIA+.

A não inclusão de artigos que versem sobre a atenção à saúde para travestis e transexuais em estados da região Norte justifica-se pelo fato de que os estudos existentes não atenderam os critérios de inclusão adotados na revisão sistemática, ou não possuíam baixo risco de viés, conforme o *checklist* utilizado para a avaliação da qualidade metodológica dos artigos^17^.

Já a escolha pela exclusão dos artigos que abordavam a atenção à saúde para travestis e transexuais em conjunto com a atenção à saúde para as demais pessoas do grupo LGBTTQIA+, justifica-se em virtude da Política Nacional de Saúde Integral LGBT reconhecer parcialmente as necessidades específicas e identitárias de travestis e transexuais, pois, apesar da existência de intercruzamentos entre as questões de gênero e de sexualidade, elas demandam análises e investimentos diferentes.

Nesse sentido, seria importante efetivar novas políticas e programas específicos para a saúde de travestis e transexuais, priorizando o enfrentamento às vulnerabilidades enfrentadas por essas pessoas^11 , 45^. No entanto, essas decisões podem ter limitado os resultados da revisão sistemática, pois é esperado que os aspectos sobre travestis e transexuais sejam abordados conjuntamente com os relacionados às pessoas lésbicas, gays, bissexuais, não-binárias etc., por causa da existência de uma política de saúde única para toda a população LGBTTQIA+ do Brasil.
